# Chemistry and Antiviral Activity of *Arrabidaea pulchra* (Bignoniaceae)

**DOI:** 10.3390/molecules18089919

**Published:** 2013-08-16

**Authors:** Geraldo Célio Brandão, Erna G. Kroon, Danielle E.R. Souza, José D. Souza Filho, Alaíde Braga Oliveira

**Affiliations:** 1 Faculdade de Farmácia, Departamento de Produtos Farmacêuticos, Universidade Federal de Minas Gerais, Av. Antônio Carlos, 6627, CEP 31.270-901, Belo Horizonte, MG, Brazil; 2 Escola de Farmácia, Departamento de Farmácia, Universidade Federal de Ouro Preto, Rua Costa Sena, 171, CEP 35.400-000, Ouro Preto, MG, Brazil; 3 Departamento de Microbiologia, ICB, Universidade Federal de Minas Gerais, Av. Antônio Carlos, 6627, CEP 31.270-901, Belo Horizonte, MG, Brazil; 4 Departamento de Química, ICEX, Universidade Federal de Minas Gerais, Av. Antônio Carlos, 6627, CEP 31.270-901, Belo Horizonte, MG, Brazil

**Keywords:** antiviral activity, dengue virus, *Arrabidaea pulchra*, verbascoside, caffeoylcallerianin

## Abstract

The aim of the present work was to carry out a bioguided isolation of antiviral chemical constituents from an ethanol extract of leaves from *Arrabidaea pulchra* (Cham.) Sandwith (EEAPL) that had shown *in vitro* activity in a previous screening using DNA and RNA viruses. The activity of EEPAL was evaluated against the DNA viruses Human herpesvirus 1 (HSV-1) and Vaccinia virus Western Reserve (VACV-WR) as well as against the RNA viruses Murine encephalomyocarditis virus (EMCV), and Dengue virus 2 (DENV-2) by the 3-(4,5-dimethylthiazol-2-yl)-2,5-diphenyltetrazolium bromide (MTT) colorimetric assay. Cytotoxicity was determined in LLCMK_2_ and Vero cells and the Selectivity Indexes (SI) were calculated. The most potent effect was observed against DENV-2 (EC_50_ 46.8 ± 1.6 µg mL^−1^; SI 2.7). For HSV-1 and VACV-WR EC_50_ values > 200 µg mL^−1^ were determined, while no inhibition of the cytopathic effect was observed with EMCV. Bioguided fractionation of EEAPL by partition between immiscible solvents followed by chromatography over a Sephadex LH20 column afforded two arylpropanoid glycosides, verbascoside (**AP 1**) and caffeoylcalleryanin (**AP 2**), along with a terpenoid, ursolic acid (**AP 3**). **AP 1** and **AP 3** exhibited similar anti-DENV-2 profiles, with SI values of 3.8 and 3.1, respectively, while **AP 2** was the most effective anti-DENV-2 constituent, with a SI of 20.0. Our results show that *A. pulchra* leaves ethanol extract (EEAPL) affords compounds with antiviral activity, mainly against DENV-2.

## 1. Introduction

Viral infections represent a current problem in industrialized and developing countries, accounting for severe damages to human health and economic losses in livestock. Plants afford an extensive chemical diversity and represent rich and renewable sources of natural products with promising biological activities. Antivirals of ethnomedicinal origin are of great interest and have been widely explored [1]. Antiviral compounds can block or inhibit virus replication by interfering with virus attachment to cells, interfering with viral enzymes or suspending viral genome replication. To be effective, compounds working at each of these steps within the virus replication cycle must target the viral process, while avoiding similar ongoing cellular processes. Viruses of different families present different structures and replication schemes, and consequently offer different potential molecular targets [2].

Some viral diseases, such as dengue, which is considered the most important human arboviral disease, have a high impact in public health. It is estimated that 100 million cases occur every year around the World. The need for a safe and efficient approach either for treatment or prevention for virus infections has been a global priority, as neither an effective drug nor a vaccine for human use is available. However, investigations against dengue viruses have started in the previous decade and opportunities for the development of anti-dengue drugs have been pointed out by the WHO [3].

The genus *Arrabidaea* belongs to the tribe Bignonieae (family *Bignoniaceae*), a large clade of neotropical lianas occurring in Central America, Amazonia, the Atlantic forests of eastern Brazil, and the open dry forests and savannas of Argentina, Bolivia, Brazil, and Paraguay [4,5]. The *Bignoniaceae* family belongs to the order Lamiales, comprising eight tribes and 104 genera, with about 860 species which occur in the tropical regions, predominantely in the neotropics [6]. In Brazil, the species *A. pulchra* (Cham.) Sandwith occurs in the “cerrado” biome (savannas), in the states of Minas Gerais, São Paulo and Mato Grosso [7]. A recent publication reports the presence of dihydroxybenzaldehyde, *p-*coumaric acid, *p*-hydroxybenzoic acid, ursolic acid, and oleanolic acid in this species [8].

Some *Arrabidaea* species are reported as anti-inflammatory, astringent, anti-syphilitic, and are used in different South American countries for the treatment of diarrhea, leucorrhea, anemia, leukaemia and skin diseases [9]. We have previously reported the antiviral activity of an *A. pulchra* ethanol leaves extract [10]. We report here the fractionation of this extract aimed at isolating its antiviral constituents. The work was guided was guided by *in vitro* assays against the DNA viruses Human herpes virus 1 (HSV-1) and Vaccinia virus Western Reserve (VACV-WR), as well as the RNA viruses Dengue virus 2 (DENV-2) and Murine encephalomyocarditis virus (EMCV).

## 2. Results

**Figure 1:**
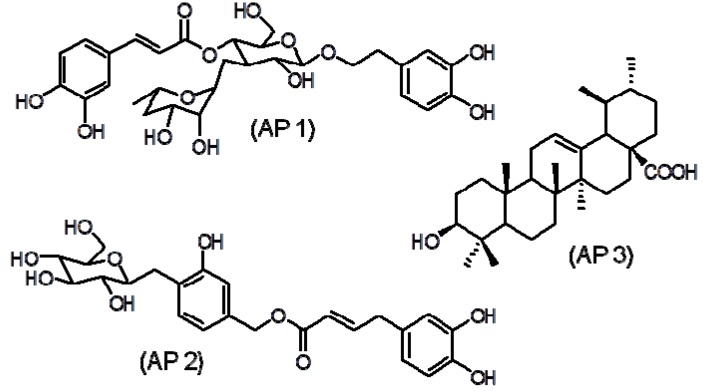
Chemical structures of verbascoside (**AP 1**), caffeoylcalleryanin (**AP 2**) and ursolic acid (**AP 3**).

Preliminary fractionation of EEAPL by sequential extractions of its aqueous methanol solution with immiscible solvents (dichloromethane, ethyl acetate and *n*-butanol) led to four fractions: dichloromethane fraction (APDL), ethyl acetate fraction (APEL), n-butanol fraction (APBL) and aqueous fraction (APAL). APDL showed good activity against HSV-1 and DENV-2, with EC_50_ values of 18.6 ± 0.9 µg/mL and 15.4 ± 2.1 µg/mL, respectively. APEL was weakly active against HSV-1 (EC50 121.9 ± 9.8 µg/mL) and disclosed good activity against VACV (EC_50_ 18.4 ± 1.9 μg/mL) and DENV-2 (EC_50_ 12.2 ± 1.6 µg/mL), with SI values > 10 in these two last viruses. Chromatography of APEL over a Sephadex LH20 column led to the isolation of verbascoside (**AP 1**, 33 mg) and caffeoylcalleryanin (**AP 2**, 23 mg). APDL (1.4 g) was exhaustively extracted with diethyl ether affording ursolic acid (**AP 3**, 203 mg). Identification of the isolated compounds was based on spectrometric analyses (UV, IR, ^1^H- and ^13^C-NMR and MS) and comparison with literature data [11,12]. The chemical structures are shown in Figure 1 and antiviral activity in Table 1.

**Figure 2:**
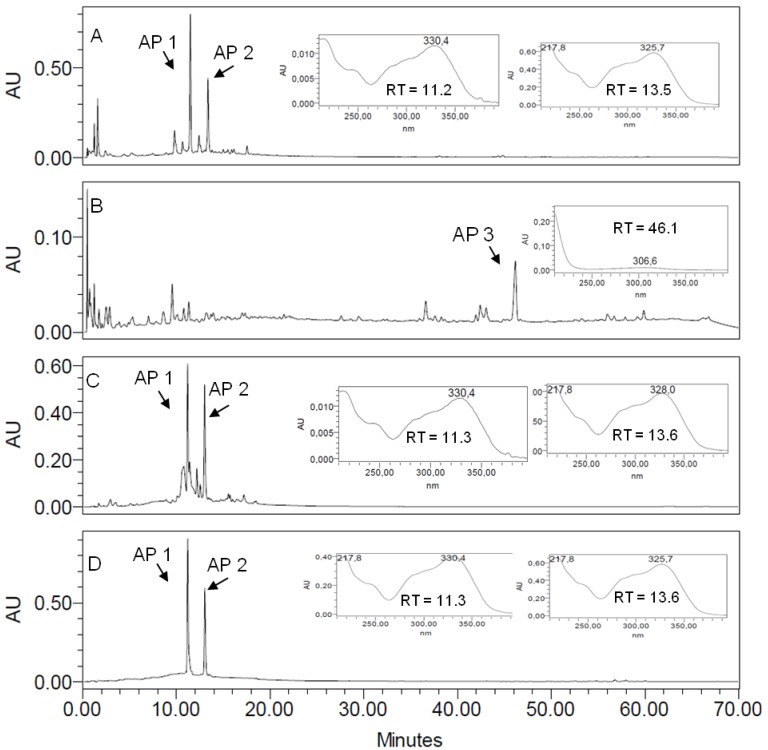
RP-HPLC-DAD fingerprints for the crude ethanol extract from *Arrabidaea pulchra* leaves (EEAPL) (**A**), APDL with **AP 3** (RT = 46.1 min UV spectrum registered online detection 220 nm (**B**), APEF with UV spectra registered online for peaks corresponding to **AP 1** (RT = 11.3 min) and **AP 2** (RT = 13.6 min) (**C**) and for a mixture of the isolated arylpropanoids verbascoside (**AP 1**, RT 11.3 min) and caffeoylcallerianin (**AP 2**, RT 13.6 min) (**D**). Detection: 350 nm. Chromatographic conditions: see Experimental Section.

**Figure 3:**
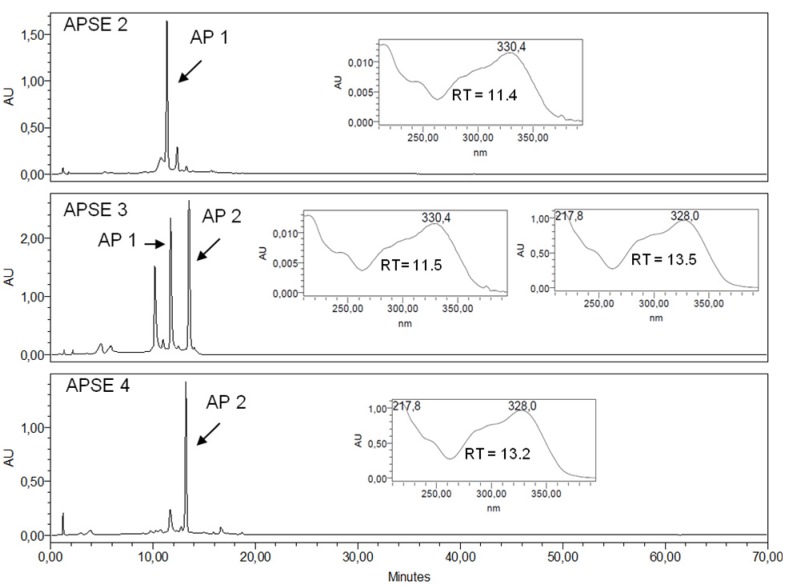
RP-HPLC-DAD profile of Sephadex LH 20 fractions APSE 2, APSE 3, APSE 4 with UV spectra registered online for peaks corresponding to **AP 1** and to **AP 2**. RT: Retention Time. Detection: 350 nm. Chromatographic conditions: see Experimental Section.

EEAPL, APDL and APEL were characterized by their RP-HPLC-DAD fingerprints. Peaks at RT 11.3 and 13.6 min in the chromatograms of EEAPL and APEL, with detection at 350 nm (Figure 2A,C), correspond to **AP 1** and **AP 2**, respectively, and both of them showed UV spectra characteristic of cinnamic acid esters. Ursolic acid (**AP 3**) was related to the peak with RT 46.1 min in the chromatographic fingerprint of APDL with detection at 220 nm and the corresponding online UV spectrum is typical of a non-conjugated alkene (Figure 2B). Figure 2 shows that **AP 1** and **AP 2** are the major constituents of APEL. Fractions APSE2, APSE3 and APSE4, which were obtained from APEL by chromatography over a Sephadex LH20 column, have quite distinct fingerprints (Figure 3). **AP-1** is concentrated in APSE2, **AP 2** is the major component in APSE4 and APSE3 contains similar concentrations of these two arylpropanoid glycosides. EEAPL, APDL, APEL fractions derived from the Sephadex LH20 column chromatography of APEL (APSE2 to APSE7), and the isolated compounds (**AP 1**, **AP 2** and **AP 3**) were assayed against HSV-1, VACV-WR, EMCV and DENV-2 (Table 1).

The ethanol extract from leaves of *A. pulchra* (EEAPL) did not show activity against EMCV, and revealed a low antiviral activity against HSV-1 (235.3 ± 9.7 μg/mL, SI > 2.21) and VACV-WR (245.2 ± 13.4 μg/mL, SI > 2.0), while good activity against DENV-2 was observed (EC_50_ 46.8 ± 1.6 μg/mL, SI 2.7) (Table 1). The dichloromethane fraction (APDL) was inactive against HSV-1 and EMCV and showed good activity against VACV and DENV-2 (EC_50_ 18.6 ± 0.9 μg/mL, SI 1.3 and 15.4 ± 2.1 μg/mL SI 1.3, respectively) but it showed high cytotoxicity against both Vero and LLCMK_2_ cells, what resulted in low SI values for these viruses. APEL was the only fraction showing activity against HSV-1 (EC_50_ 121.9 ± 9.8 μg/mL, SI > 1.6), although it was more potent against VACV-WR (EC_50_ 18.4 ± 1.9 μg/mL, SI > 10.9) and DENV-2 (EC_50_ 12.2 ± 1.6 μg/mL, SI > 16.3). Fractions APSE 2, 3 and 4 that were obtained from cromatography of APEL on a Sephadex LH20 column disclosed good activity against VAC-WR (EC_50_ 15.9 ± 2.3 μg/mL, SI > 12.3 to EC_50_ 18.1 ± 3.4 μg/mL, SI > 11.0) and against DENV-2 (EC_50_ 8.8 ± 0.7 μg/mL, SI > 22.7 to EC_50_ 11.9 ± 1.5 μg/mL, SI > 16.8).

Compounds **AP 1**, **2** and **3** were not active towards VAC-WR and EMCV, and **AP 3** showed a low activity towards HSV-1. The three compounds disclosed good activity against DENV-2, although the SI values are low for AP 1 and AP 3, as a consequence of their toxicity to LLCMK_2_ cells. **AP 2** was the most potent one, with SI > 10 (EC_50_ 2.8 ± 0.4 μg/mL, SI 20.0, Table 1).

**Figure 4:**
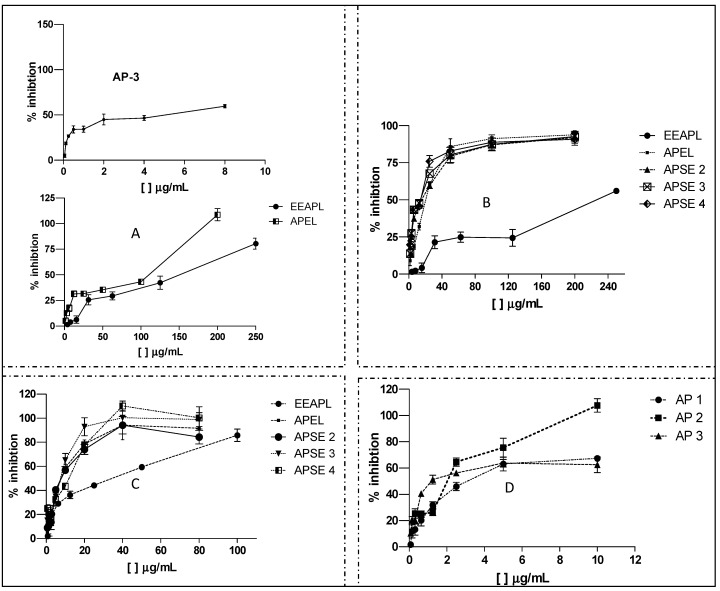
Dose-response curves for antiviral activity of ethanol extract, fractions, and constituents of *Arrabidaea pulchra* leaves. (**A**) EEAPL, APEL and **AP 3** against HSV-1; (**B**) EEAPL, APEL, APSE 2–4 against VAC-WR; (**C**) EEAPL, APEL, APSE 2–4 against DENV-2; (**D**) **AP 1**, **AP 2**, **AP 3** against DENV-2.

Dose-response curves for the active samples are shown in Figure 4. Figure 4A shows that EEAPL and APEL were very active against HSV-1 (virus inhibition > 75%) at concentrations higher than 150 μg/mL, while **AP 3** was active at concentrations as low 2 μg/mL, but with only 50% of virus inhibition. EEAPL was more active against VACV-WR at concentrations of 240 μg/mL, inhibiting 50% of the virus, while the fraction APEL and the Sephadex LH20 fractions APSE2-4, derived from it, have shown enhanced antiviral effects, with inhibition > 75% at concentrations of approximately 40 μg/mL.

**Table 1:** Cytotoxicity (CC_50_, Vero and LLCMK_2_ cells), *in vitro* antiviral activity (EC_50_), selectivity index (SI) for ethanol extract from *Arabidaea pulchra* leaves(EEAPL), fractions APDL, APEL, APSE2-4 and compounds **AP 1**–**3**.

Extract Fractions Compounds	Vero cells CC_50_µg/mL	LLCMK_2_ cells CC_50_µg/mL	^a^ HSV-1 EC_50_ µg/mL	SI	^b^ VACV-WR EC_50_ µg/mL	SI	^c^ EMCV EC_50_ µg/mL	^d^ DENV-2 EC_50_ µg/mL	SI
EEAPL	>500	124.4 ± 0.8	235.3 ± 9.7	>2.1	245.2 ± 13.4	>2.0	NA	46.8 ± 1.6	2.7
APDL	25.0 ± 0.3	19.6 ± 1.7	NA		18.6 ± 0.9	1.3	NA	15.4 ± 2.1	1.3
APEL	>200	>200	121.9 ± 9.8	>1.6	18.4 ± 1.9	>10.9	NA	12.2 ± 1.6	>16.3
APSE2	>200	>200	NA		16.7 ± 1.8	>12.0	NA	10.6 ± 2.1	>18.9
APSE3	>200	>200	NA		18.1 ± 3.4	>11.0	NA	11.9 ± 1.5	>16.8
APSE4	>200	>200	NA		15.9 ± 2.3	>12.3	NA	8.8 ± 0.7	>22.7
AP 1	>200	12.9 ± 1.1	NA		NA		NA	3.4 ± 0.4	3.8
AP 2	>200	56.1 ± 2.4	NA		NA		NA	2.8 ± 0.4	20.0
AP 3	8.1 ± 0.9	9.9 ± 1.3	6.2 ± 0.1	1.6	NA		NA	3.2 + 0.6	3.1
Acyclovir			^a^ 40						
α-2a Interferon					^ef^ 2.5 × 10^2^		^ef^ 1.5 × 10^2^	^ef^ 2.5 × 10^3^	

SI, selective index; ^a^ viral titer TCID_50_/mL 2.5 × 10^6^ in 48 h; ^b^ viral titer TCID_50_/mL 1.0 × 10^6^ in 48 h; ^c^ viral titer TCID_50_/mL 1.0 × 10^6^ in 48 h; ^d^ viral titer TCID_50_/mL 1.0 × 10^4^ in 72 h; NA, no activity in the assayed concentrations; ^e^ 80 to 100% inhibition of cytopathic effect; ^f^ concentration in UI/mL; EEAPL, ethanol extract from *Arrabidaea pulchra* leaves; APDF, *Arrabidaea pulchra* dichloromethane fraction; APEL, *Arrabidaea pulchra* ethyl acetate fraction; **AP 1**, verbascoside; **AP 2**, caffeoylcallerianin; **AP 3** ursolic acid; APSE2-4, *Arrabidaea pulchra* fractions from chromatography of APEL over Sephadex LH 20^®^ column.

The strongest EEAPL antiviral effect was towards DENV-2 with 80% inhibition at 100 μg/mL. APEL and APSE2-4 caused approximately 100% inhibition at concentrations of 40 μg/mL. Finally, the highest potency of **AP 2** against DENV-2 was demonstrated by the dose-response curves of Figure 4D, in which 100% of virus inhibition was observed at 10 μg/mL.

## 3. Discussion

According to our previously published results [10], the antiviral profile of the ethanol extract of the leaves of *A. pulchra* (EEAPL) is inactive towards EMCV, shows low activity against HSV-1 (235.3 ± 9.7 μg/mL, SI > 2.21) and VACV-WR (245.2 ± 13.4 μg/mL, SI > 2.0) and good activity towards DENV-2 (EC_50_ 46.8 ± 1.6 μg/mL), although with a low SI (2.7, against LLCMK_2_ cells). (Table 1). Based on the EC_50_ value lower than 100 μg/mL as determined for EEAPL, the limit we established for a plant extract to be considered of interest for a bioguided fractionation, this extract was initially submitted to a liquid-liquid partition aiming to promote the separation of the EEPAL constituents according to their polarity/solubility. The dicloromethane fraction (APDL) inhibited the viral replication of VACV-WR and DENV-2, showing EC_50_ values of 18.6 ± 0.9 μg/mL (VACV-WR) and 15.4 ± 2.1 μg/mL (DENV-2), both of them with low SI (1.3). On the other hand, ursolic acid (**AP 3**), which was isolated from APDL, was inactive against VACV-WR, but has shown activity against HSV-1 and DENV-2 (EC_50_ 6.2 ± 0.1 μg/mL and 3.2 + 0.6 μg/mL, respectively) although with low SI values (1.6 and 3.1, respectively), due to its cytotoxicity to Vero and LLCMk2 cells (8.1 ± 0.9 and 9.9 ± 1.3 μg/mL, respectively). APEL showed a better profile than APDL for both VACV and DENV-2, with good antiviral activity (EC_50_ < 20 μg/mL) and SI > 10 and, therefore, it was submitted to chromatography over a Sephadex LH20 column aiming at separation of its active constituents. Three fractions (APSE2-4) out of the seven fractions obtained have shown good activity against VACV-WR and DENV-2 with SI > 10, for both of these viruses, and particularly against DENV-2 (SI > 22.7). APSE 2 led to the isolation of verbascoside (**AP 1**) and caffeoylcallerianin (**AP 2**) from APSE4. **AP 1** and **AP 2** have inhibited the replication of DENV-2 with EC_50_ values of 3.4 μg/mL and 2.8 μg/mL, respectively, but **AP 2** was less cytotoxic to LLCMk2 cells, thus resulting in a more favorable SI (20.0), while for **AP 1** the SI value (3.8) is approximately five times lower. Indeed, the cytotoxicity of ursolic acid is recognized. It is reported to be involved in endoplasmic reticulum stress pathway [13] targeting apoptosis and its potent cancer-preventive activity and great cancer therapeutic potential have been pointed out [14]. None of the tested samples inhibited the multiplication of EMCV. EEAPL, fractions derived from it and isolated compounds have generally shown low cytotoxicity, except APDL and **AP 1** with CC_50_ in the range of 19.6 μg/mL for LLCMK_2_ cells and 25.9 μg/mL for Vero cells. Concerning the effects against DENV-2, it is interesting to note that APEL, which contains similar contents of **AP 1** and **AP 2** (Figure 2), has shown an enhancement of the antiviral activity (SI > 16.3) in relation to the crude ethanol extract (EEAPL, SI 2.7) while APSE4 (SI > 22.7), containing predominantly **AP-2** (Figure 3), was closer to the value for this compound (SI 20.0). Caffeoylcalleryanin (**AP 2**) was first isolated in 1968 from *Pyrus calleryana* (Rosaceae) [12], however this is the first report on its isolation from a Bignoniaceous species, as well as on its antiviral activity.

Verbascoside (**AP** 1, synonyms: acteoside, kusaginin) is a phenylethanoid glycoside isolated from various plant species that displays an interesting spectrum of biological activities [15]. In the genus *Arrabidaea*, verbascoside was isolated from *A. harleyi* [11] and its occurrence has been reported in some other genera of the family. It was also isolated from *Markhamia lutea* (Bignoniaceae) and has shown a potent antiviral effect against respiratory syncytial virus (RSV, EC_50_ 0.80 µg/mL) [16]. Recently the activity against HSV-1 (EC_50_ 58.0 µg/mL) and HSV-2 (EC_50_ 8.9 µg/mL) was reported for this compound [17]. However, in the present work, no effect was observed for verbascoside against this virus. Our result is in agreement with a previous report in which this compound had not shown anti-herpes activity either [16] and these contradictory results might be related to different degrees of virulence of the strains used in the assays.

Ursolic acid (**AP 3**), a pentacyclictriterpene acid, is frequently found in several plant taxa and has shown a very wide biological activity profile [18]. We have recently described its activity against the same strain of HSV-1 when investigating the antiviral activity of *Distictella elongata* (Vahl) Urb. (Bignoniaceae) [19]. Terpenoids possess a broad spectrum of antiviral activity against diverse virus families, as reported in a review on antivirals of ethnomedicinal origin that unfortunately does not report the cytotoxicity of the compounds. Ursolic acid, related triterpenoids and their derivatives isolated from many plants inhibit HIV-1 protease and the stability of the gp120/gp41 complex. The mechanisms of antiviral action of this group of triterpenoids have been shown to involve inhibition of virus entry, of protease and of replication [1].

Verbascoside (**AP 1**) and caffeoylcalleryanin (**AP 2**) belong to the group of naturally occurring polyphenols that are known as phenylethanoid glycosides, supporting each one a moiety of glucose esterified by caffeic acid and bonded to an ethylcatechol unity by an ether linkage. The polyphenols often showed virucidal effects in several viral systems by attaching to the proteins and/or host cell surfaces, resulting in reduction or prevention of viral adsorption; inhibition of reverse transcriptase (RTase) of HIV-1 and RNA polymerase of influenza virus. The *ortho*-dihydroxy systems are probably responsible for polyphenols’ antiviral activity [1].

In light of our results, further investigations are needed to understand the mechanisms of the antiviral activity, especially of caffeoylcalleryanin, which shows the lowest toxicity in LLCMK_2_ cells of the tested compounds and was active only in DENV-2. Further assays are also needed to investigate virucidal activity and targets in the viral replication cycle.

## 4. Experimental

### 4.1. Plant Material

The leaves of *A. pulchra* were collected in Belo Horizonte, Minas Gerais, Brazil. The plants were taxonomically identified by Dr. J.A. Lombardi, Departamento de Botânica, Instituto de Biociências, UNESP, Rio Claro, Brazil. A voucher specimen was deposited at the BHCB/UFMG, Belo Horizonte, Minas Gerais, Brazil, under the number 21573.

### 4.2. Extraction and Isolation

**Figure 5:**
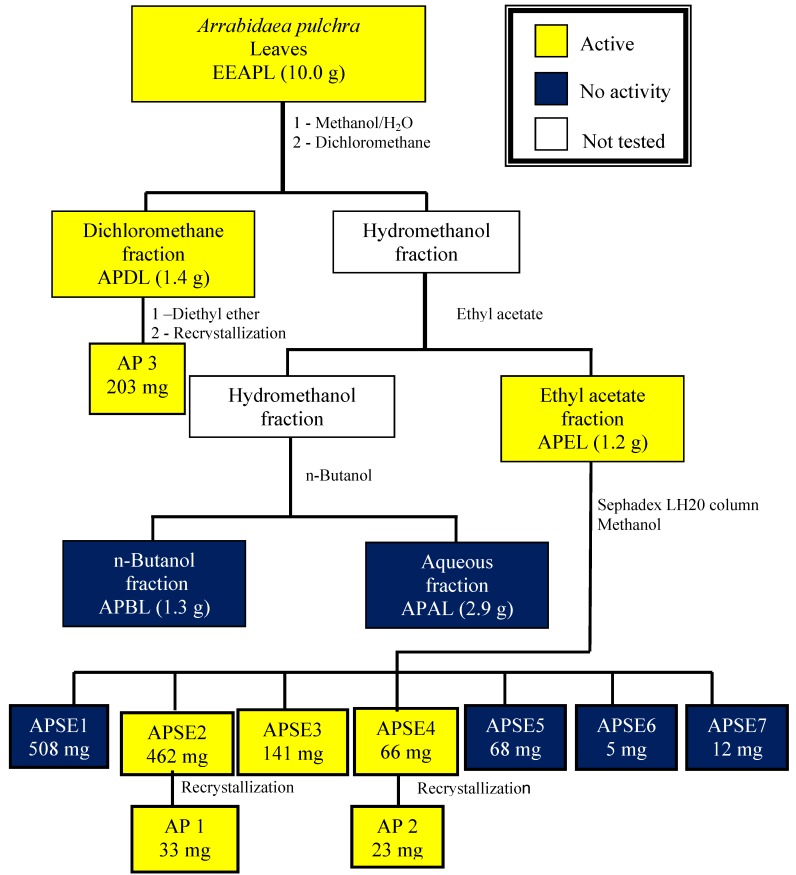
Bioguided fractionation of the ethanol extract from *Arrabidaea pulchra* leaves.

The leaves (129.6 g) were dried in a ventilated oven at 40 °C, for 72 h, ground and exhaustively extracted by percolation with 96% ethanol (5 × 250 mL) at room temperature for 48 h each time. A crude dark green ethanol extract residue (EEAPL, 30.8 g) was obtained in a rotatory evaporator under reduced pressure at 50 °C. A portion of EEAPL (10.0 g) was dissolved in methanol-H_2_O (7:3) and this solution was submitted to successive extractions with immiscible solvents affording the corresponding dichloromethane (APDL, 1.4 g), ethyl acetate (APEL, 1.5 g), *n*-butanol (APBL, 1.3 g) fractions and the final aqueous fraction (APAL, 2.9 g). Chromatography of APEL (1.2 g) through a Sephadex LH 20^®^ column eluted with methanol, led to 66 fractions of 10 mL each, which were combined according to their TLC profiles, affording a total of seven fractions (APSE1-7). Recrystallization of fractions APSE2 and APSE4 from methanol yielded **AP 1** (33 mg) and **AP** 2 (23 mg), respectively. APDL was exhaustively extracted with diethyl ether that was concentrated to a residue (430 mg) which was recrystallized from ethanol to give ursolic acid (**AP 3**, 203 mg). The fractionation process was undertaken as shown in Figure 5.

### 4.3. Structural Determination

AP 1, AP 2 and AP 3 were identified on the basis of spectral analyses and comparison with literature data. ^1^H-NMR, ^13^C-NMR, NOESY, TOCSY, HSQC; HMBC spectra were registered in DMSO-*d_6_* with TMS as internal standard and were recorded on a Bruker Advance DPX400 instrument. Chemical shifts are given as δ (ppm). LC-MS were obtained by electrospray ionization mass spectrometry (ESI-MS) in an Esquire 3000 Plus Bruker Daltonics equipment, Capillary: 4000 V, Nebulizer: 27 psi, Dry Gas: 7.0 L/min, Dry Temp: 320 °C, mass flux 100 uL/min, in the Central Analítica, Instituto de Química, Universidade de São Paulo, São Paulo, SP, Brazil.

### 4.4. Spectral Data

*Verbascoside* (**AP 1**). Light yellow powder. MP 145–148 °C, Lit. 149–151 °C [20]. UV (MeOH): λ_max_ (log ε) 219.5 (4.6), 250 (sh), 290 (sh), 332.0 (4.6) nm. IR: u_max_ 3327, 1660, 1599, 1515, 1445, 1368, 1258, 1157, 1113, 1063, 1050, 853, 809, 784 cm^−1^. ^1^H-NMR (DMSO-*d_6_*): δ = 7.02 (1H, *J* = 2.0 Hz, H-2′′′), 6.97 (1H, dd, *J* = 2.0 and 8.0 Hz; H-6′′′), 6.76 (1H, d, *J* = 8.0; H-5′′′), 6.64 (1H, dd, *J* = 8.0, H-5), 6.63 (1H, s, H-2), 6.49 (1H, dd, *J* = 2.0 and 8.0 Hz, H-6), 7.46 (1H, d, *J* = 16.0 Hz, H-7′′′), 6.19 (1H, d, *J* = 16.0 Hz; H-8′′′), 5.03 (1H, d, *J* = 8.0 Hz, H-1′), 4.35 (1H, d, *J* = 8.0 Hz, H-1′′). ^13^C-NMR (DMSO-*d_6_*): δ = 129.1 (C1), 116.3 (C2), 145.0 (C3), 143.5 (C4), 115.4 (C5), 119.5 (C6), 35.5 (C7), 69.1 (C8), 101.2 (C1′), 75.0 (C2′), 79.5 (C3′), 69.6 (C4′), 72.1 (C5′), 61.2 (C6′), 102.3 (C1′′), 70.9 (C2′′), 70.7 (C3′′), 71.0 (C4′′), 69.2 (C5′′), 18.6 (C6′′), 125.5 (C1′′′), 114.7 (C2′′′), 145.5 (C3′′′), 148.4 (C4′′′), 115.8 (C5′′′), 121.3 (C6′′′), 145.0 (C7′′′), 113.6 (C8′′′), 165.7 (C9′′′). ESI-MS: found *m/z* 647.2 [M+Na]; calculated for C_29_H_35_O_15_Na *m/z* 647.

*Caffeoylcalleryanin* (**AP 2**). Light yellow powder. MP 167–171 °C. UV (MeOH): λ_max_ (log ε) 217 (4.3), 250 (sh), 286.5 (4.1), 300 (sh), 329.5 (4.2) nm. IR: u_max_ 3270, 1660, 1599, 1510, 1443, 1353, 1253, 1175, 1150, 1035, 803 cm^−1^. **^1^**H-NMR (DMSO-*d_6_*): δ = 7.07 (1H, d, *J* = 2.0 Hz; H-2′′), 7.02 (1H, dd, *J* = 2.0 and 8.0 Hz; H-6′′), 6.78 (1H, d, *J* = 8.0; H-5′′), 7.12 (1H, dd, *J* = 8.0, H-5), 6.89 (1H, d, *J* = 2.0 Hz, H-2), 6.81 (1H, dd, *J* = 2.0 and 8.0 Hz, H-6), 7.52 (1H, d, *J* = 16.0 Hz, H-7′′′), 6.32 (1H, d, *J* = 16.0 Hz; H-8′′′), 4.71 (1H, d, *J* = 7.2 Hz, H-1′), 3.2–3,84 (H-2′, H-3′,H-4′, H-5′,H-6′a e 6′b, m). ^13^C-NMR (DMSO-*d_6_*): δ = 131.0 (C1), 115.9 (C2), 145.1 (C3), 146.8 (C4), 116.7 (C5), 119.3 (C6), 65.1 (C7), 102.3 (C1′), 75.9 (C2′), 77.2 (C3′), 69.6 (C4′), 73.3 (C5′), 60.8 (C6′), 125.4 (C1′′), 114.8 (C2′′), 145.6 (C3′′), 148.6 (C4′′), 115.7 (C5′′), 121.4 (C6′′), 145.4 (C7′′), 113.7 (C8′′), 166.4 (C9′′). ESI-MS: found *m/z* 487.1 [M+Na]; calculated for C_22_H_24_O_11_Na *m/z* 487.0.

*Ursolic acid* (**AP 3**). Colourless crystals. MP 282–284 °C, Lit 285–287 °C [21]. IR: u_max_ 3392, 2973, 2852, 1687, 1620, 1456, 1387, 1259, 1089, 867, 798; ^1^H-NMR (DMSO-*d_6_*): 5.23 (bs, 1H, H-12), 3.0 (m, 1H, H-3), 2.11 (d, 1H, J11.2, H-18), 0.69; 0.71; 0.87; 0.91; 1.08; 1.29 (6s, 6xCH_3_). ^13^C-NMR (DMSO-*d_6_*): δ 38.1 (C-1), 26.9 (C-2), 76.7 (C-3), 32.6 (C-4), 54.7 (C-5), 17.9 (C-6), 36.4 (C-7), 39.5 (C-8), 46.9 (C-9), 38.3 (C-10), 22.8 (C-11), 124.5 (C-12), 138.1 (C-13), 41.6 (C-14), 27.4 (C-15), 23.7 (16), 46.7 (C-17), 52.3 (C-18), 38.4 (C-19), 38.4 (C-20), 30.1 (C-21), 36.2 (C-22), 28.2 (C-23), 15.1 (C-22), 28.2 (C-23), 15.1 (C-24), 16.0 (C-25), 16.8 (C-26), 23.2 (C-27), 178.5 (C-28), 16.9 (C-29), 21.0 (C-30). EI-MS *m/z* (%): 43 (100), 55 (56), 67 (26), 68 (36), 79 (23), 81 (30), 91 (23), 93 (23), 95 (26), 105 (24), 107 (20), 109 (16), 133 (44), 147 (17), 163 (7), 175 (9), 189 (15), 203 (31), 207 (26), 248 (76), 249 (13), 456 (M^+^) (2).

### 4.5. HPLC Analyses

Fingerprints were registered by RP-HPLC-DAD on a Waters 2695 apparatus equipped with a UV-DAD detector (Waters 2996). A LiChrospher 100 RP-18 column (5 μm, 250 × 4 mm i.d.; Merck, Darmstadt, Germany) was employed at 40 °C, flow rate of 1.0 mL/min and detection at wavelengths of 220, 280 and 350 nm. To an aliquot (10.0 mg) dried extract/fractions and (1.0 mg) of each of the isolated compounds (AP 1, AP 2 and AP 3), HPLC grade methanol was added and the mixture was dissolved by sonication in an ultrasound bath for 15 min, followed by centrifugation at 10,000 rpm for 10 min. The supernatant was filtered through a Millipore membrane (0.2 μm) and injected (10.0 μL) onto the equipment. Elution was carried out with a linear gradient of water (A) and acetonitrile (B) (from 5% to 95% of B in 60 min).

### 4.6. Cell Culture and Virus

Vero cells (ATCC CCL-81) and LLCMK_2_ cells were cultured in Dulbecco’s modified Eagle’s medium (DMEM, Cultilab, Campinas, SP, Brazil) at 37 °C, in 5% CO_2_ atmosphere, supplemented with 5% fetal bovine serum, 50 µg/mL gentamicin, 100 U/mL penicillin and 5 µg/mL amphotericin B. HSV-1 was obtained from the collection of Laboratório de Virus, UFMG, Belo Horizonte, Brazil. DENV-2, EMCV and VACV-WR were kindly donated by Dr. L. Figueiredo (USP, Ribeirão Preto, Brazil), Dr. I. Kerr (London Research Institute, London, UK) and Dr. C. Jungwirth (University of Würzburg, Würzburg, Germany), respectively. The viruses were titrated by TCID_50_ in Vero cells [22] and the titers were 2.5 × 10^6^; 1.0 × 10^6^; 1.0 × 10^6^ and 1.0 × 10^4^ TCID_50_/mL, respectively, for HSV-1, EMCV, VACV-WR and DENV-2 virus.

### 4.7. Cytotoxicity Assay

Vero and LLCMK_2_ cells were exposed to different concentrations of extracts/fractions/compounds for 48 and 72 h. After incubation, cell viability was assessed by the 3-(4,5-dimethylthiazol-2-yl)-2,5-diphenyltetrazolium bromide (MTT, Merck) assay at a concentration of 2 mg mL^−1^ in PBS [23]. Each sample was assayed in four replicates for concentrations ranging from 500 to 0.125 µg mL^−1^. The cytotoxicity of each sample was expressed as CC_50_, i.e. the concentration of sample that inhibited cell growth by 50%.

### 4.8. Antiviral Assays

The antiviral activity (EC_50_) was evaluated by the MTT assay [23]. Acyclovir (Calbiochem, Merck Brasil, São Paulo, SP, Brazil) and α-2a interferon (Bergamo Brasil, São Paulo, SP, Brazil) were used as positive controls. Experiments were carried out with eight different concentrations within the inhibitory range of the samples. The 50% inhibitor concentration of the viral effect (EC_50_) for each extract, fractions, and constituents were calculated from concentration-effect-curves after no linear regression analysis (Figure 5). The selective index (SI) is defined as CC_50_ over EC_50_.

## 5. Conclusions

Our results reveal the ethanol extract of *A. pulchra* leaves (EEAPL) to be a promising source of the anti-dengue arylpropanoid glycoside derivatives verbascoside and caffeoylcallerianin, which might represent the main responsible constituents for its activity against DENV-2. Our findings are in line with the traditional use of *Arrabidaea* species as anti-infectious agents in different South American countries [9] and are of interest for the development of standardized phytomedicines [24].
